# External Validation of UDCA Response Score in Slovak and Croatian Patients with Primary Biliary Cholangitis

**DOI:** 10.1155/2021/9928065

**Published:** 2021-06-22

**Authors:** Jakub Gazda, Martin Janicko, Sylvia Drazilova, Ivica Grgurevic, Tajana Filipec Kanizaj, Tomas Koller, Beatrica Bodorovska, Maja Mijic, Ivana Mikolasevic, Ivana Knezevic Stromar, Branislav Kucinsky, Matej Gazda, Peter Jarcuska

**Affiliations:** ^1^2nd Department of Internal Medicine, Pavol Jozef Safarik University in Kosice, Louis Pasteur University Hospital, Trieda SNP 1, 040 11 Kosice, Slovakia; ^2^Internal Medicine Department, Nemocnica Poprad a.s., 05801 Poprad, Slovakia; ^3^Department of Gastroenterology, Hepatology and Clinical Nutrition, University of Zagreb School of Medicine, University Hospital Dubrava, Avenija Gojka Suska 6, Zagreb 10000, Croatia; ^4^Department of Gastroenterology, University of Zagreb School of Medicine, University Hospital Merkur, Zajceva ul. 19, Zagreb 10000, Croatia; ^5^5th Department of Internal Medicine, Subdivision of Gastroenterology and Hepatology, Comenius University Faculty of Medicine, University Hospital Bratislava, Ruzinovska 6, 826 06 Bratislava, Slovakia; ^6^Clinic of Gastroenterological Internal Medicine, Comenius University in Bratislava, University Hospital Martin, Kollarova 2, 036 59 Martin, Slovakia; ^7^Department of Gastroenterology, University Hospital Merkur, Zajceva ul. 19, Zagreb 10000, Croatia; ^8^Department of Gastroenterology, UHC Rijeka, Croatia and Faculty of Medicine, University of Rijeka, Rijeka, Croatia; ^9^Department of Gastroenterology and Hepatology, University Hospital Centre Zagreb, Kispaticeva ul. 12, Zagreb 10000, Croatia; ^10^Department of Mathematics and Theoretical Informatics, Technical University of Kosice, Bozeny Nemcovej 32, 040 01 Kosice, Slovakia

## Abstract

**Background:**

Ursodeoxycholic acid response score (URS) is a prognostic model that estimates the baseline probability of treatment response after 12 months of ursodeoxycholic acid (UDCA) therapy in patients with primary biliary cholangitis (PBC).

**Aim:**

To independently evaluate the predictive performance of the URS model.

**Methods:**

We used a cohort of Slovak and Croatian treatment-naïve PBC patients to quantify the discrimination ability using the area under receiver operating characteristic curve (AUROC) and its 95% confidence interval (CI). Furthermore, we evaluated the calibration using calibration belts. The primary outcome was treatment response after 12 months of UDCA therapy defined as values of alkaline phosphatase ≤1.67 × upper limit of normal.

**Results:**

One hundred and ninety-four patients were included. Median pretreatment age was 56 years (interquartile range 49–62). Treatment response was achieved in 79.38% of patients. AUROC of the URS was 0.81 (95% CI 0.73–0.88) and the calibration belt revealed that response rates were correctly estimated by predicted probabilities.

**Conclusion:**

Our results confirm that the URS can be used in treatment-naïve PBC patients for estimating the treatment response probability after 12 months of UDCA therapy.

## 1. Introduction

Primary biliary cholangitis (PBC) is a chronic cholestatic autoimmune liver disease. PBC incidence rates range from 0.33 to 5.8 per 100,000 inhabitants/year and prevalence rates range from 1.91 to 40.2 per 100,000 inhabitants and are increasing with time [1]. In Slovakia, annual PBC incidence rates range from 0.7 to 1.5 cases per 100,000 inhabitants/year, and the 2018 point prevalence was 14.1 cases per 100,000 inhabitants [2]. Similarly, in Croatia, PBC incidence rates range from 0.3 to 3.04 cases per 100,000 inhabitants/year and the 2017 point prevalence was 11.5 and 12.5 cases per 100,000 inhabitants in the continental and coastal regions, respectively [3]. Immunological attack on biliary epithelial cells with secondary failure of biliary transporters is, together with epigenetic mechanisms, generally considered to play a major role in the disease's pathogenesis [4]. The hallmark for diagnosis of PBC is serological positivity for antimitochondrial antibodies (AMA) [5]. Furthermore, ancillary markers anti-sp100 and anti-gp210 (antinuclear antibodies) are also used in clinical practice, because their positivity strongly suggests the diagnosis of PBC, irrespective of antimitochondrial antibody status [5]. PBC often results in end-stage liver disease and its associated complications [5]. Progression to the moderate stage occurs in about half of patients with the early stage of the disease. Subsequently, 16% of patients with the moderate stage transit to advanced PBC over a five-year period despite receiving treatment with ursodeoxycholic acid (UDCA) [6]. UDCA increases the proportion of patients with 10-year transplant-free survival by about 20%–40% compared with receiving no treatment or placebo [7, 8]. Patients who achieve treatment response to UDCA therapy in the early stage of the disease have survival rates comparable with the general population [9], and a relatively modest improvement in overall survival is related to a proportion of patients who fail to achieve treatment response. Based on the published data, treatment response is achieved in 46%–74% of all treated patients [10]. Notably, despite its suboptimal efficacy, UDCA remains the first-line treatment option for PBC. Clinical trials have shown that UDCA nonresponders benefit from the addition of either bezafibrate or obeticholic acid [11, 12]. A second-line treatment has already been conditionally approved in combination with UDCA for patients showing an inadequate response to UDCA [5]. Therefore, it is important to identify patients who would not benefit from the first-line treatment, so that they can be offered the second-line treatment whilst still in the early stage of the disease. Additionally, accurate selection of poor first-line treatment responders is also important for the recruitment to clinical trials of new drugs, so they can better demonstrate efficacy compared to UDCA, which is still the standard of care. The UDCA response score (URS) is a recently developed logistic regression model for PBC patients [13]. The URS model was designed to estimate the baseline probability of treatment response after 12 months of UDCA therapy. The authors defined treatment response as ALP < 1.67 × ULN because this was how UDCA response had been defined in clinical trials of second-line agents. The URS is a multivariable prognostic model, which explores the relationship of treatment response and the following independent variables: age at diagnosis (in years; ((age_diag_)), total bilirubin at diagnosis (in multiples of the upper limit of normal ((×ULN); (TB_diag_)), aminotransferase (either aspartate aminotransferase (ASTdiag) or alanine aminotransferase (ALTdiag)) at diagnosis (in ×ULN; (AT_diag_)), alkaline phosphatase at diagnosis (in ×ULN; (ALP_diag_)), treatment time lag (in years), and change in ALP from diagnosis to start of treatment (ΔALP). The authors used a composite variable AT, which was ALT when available; otherwise, AST was used. Depending on a patient's age at diagnosis and laboratory status only, it precludes any interrater variability in the interpretation of the results. The URS was developed on a well-defined UK-PBC cohort of patients, with good discriminatory ability in the derivation cohort (AUROC 0.87; 95% CI 0·86–0·89). The model was also externally validated on the GLOBE cohort of PBC patients in the original development study (AUROC 0.83; 95% CI 0·79–0·87). Calibration belts revealed that the model was well-calibrated on both the UK-PBC and GLOBE cohorts. A URS calculator is available online (https://www.mat.uniroma2.it/∼alenardi/URS.html).

Risk prediction models, such as the URS, can play an essential role in decision-making and future management of patients. It is imperative that these models are transferable and may be used with confidence in any population of patients with the respective medical condition [14]. However, a model might not perform as well as originally reported when it is used in clinical practice due to regional differences in patient populations. Thus, it is important that these risk prediction models are convincingly validated in external cohorts of patients prior to being applied in clinical practice [15]. Aside from the original study, the model's predictive performance has thus far only been evaluated in Japanese PBC patients [16]. In this paper, we aimed to independently evaluate the predictive performance of the URS model on a combined dataset of Slovak and Croatian PBC patients.

## 2. Methods

We performed an international multicentre retrospective validation study in a cohort of patients who were consecutively diagnosed with PBC and started UDCA treatment at ten hepatology centers in Slovakia (5) and Croatia (5) during the period from 30 June 1999 through 30 June 2019.

The exclusion criteria were as follows: (a) insufficient data for verifying the PBC diagnosis, (b) immunosuppressive or obeticholic acid treatment, (c) liver transplantation after less than 12 months of UDCA treatment, (d) patients with missing data that prevented the assessment of treatment response, and (e) patients with any of the URS predictors missing.

Local investigators completed case report forms (CRF) with on-call assistance from the study coordinators and collected pretreatment (*T*_0_) demographic and clinical information and initial UDCA dosage. To account for interlaboratory variability, TB, AST, ALT, and ALP were all transformed into a multiple of their respective ULNs. Furthermore, CRF included information on immunosuppressive treatment or obeticholic acid and history of liver transplantation status, and it also contained data necessary for evaluating treatment response after 12 months of UDCA therapy (*T*_12_). All centers used immunofluorescence technique to detect AMA, and three of them verified the AMA positivity using western immunoblotting.

Every patient was centrally evaluated for PBC diagnosis following the European Association for the Study of the Liver (EASL) recommendations [5] that states that two out of the three following criteria need to be met: (1) elevated ALP, (2a) the presence of antimitochondrial antibodies (AMA) at a titer >1 : 40 or (2b) the presence of anti-sp100/anti-gp210, and (3) histological signs after liver biopsy.

We used the same Toronto [17] treatment response definition as the one used in the original development study (ALP < 1.67 × ULN) and evaluated patients for achieving it after a 12-month course of UDCA.

The baseline UDCA response score was calculated using logistic regression formula provided by Carbone et al.:

  UDCA response score (URS) = 0.77 + 0.60 × (√TB_diag_)^−1^ – 2.73 × ln (ALP_diag_) + 0.35 × ln (AT_diag_) + 0·03 × age – 0·15 × (treatment time lag) – 0.56 × ΔALP.

Slovak and Croatian patients included in the final analyses received UDCA immediately following the diagnosis of PBC (*T*_0_ = *T*_diag_). Therefore, we substituted TB_T0_ for TB_diag_, ALP_T0_ for ALP_diag_, and AT_T0_ for AT_diag_ and set both the treatment time lag and ΔALP to 0. We used ALT in the place of the composite AT variable.

We estimated that the pretreatment probability of treatment response achievement after 12 months of UDCA therapy is as follows:

  Probability = Exp (URS)/(1 + EXP (URS))

The study protocol is in accordance with the 1964 Declaration of Helsinki and its later amendments and with the principles of good clinical practice. The study protocol was approved by the Ethical Committee of Poprad Hospital, a.s., on 5 May 2019. Due to the retrospective nature of data collection and the complete anonymity of the records even from the principal investigator (only local investigators responsible for the standard of care could identify the patients), the committee waived the need for specific patients' informed consent. All authors had access to the study data and have reviewed and approved the final manuscript.

## 3. Statistical Analyses

We did not perform formal sample size calculations. However, all eligible data available for the URS model validation were considered to maximize the power and generalizability of the results.

We reported the clinical and demographic characteristics of patients using medians and interquartile ranges (IQR) for the continuous variables and absolute counts and percentages for the categorical variables. Additionally, we used boxplots to visualize the distribution of the continuous variables. Mann–Whitney and *χ*^2^ tests were used to evaluate the statistical significance of differences in continuous and categorical variables, respectively. Furthermore, we compared the patients' characteristics with those from the derivation (UK-PBC) cohort. However, it was impossible to test the significance of differences in the continuous variables, given that only summary statistics (medians and interquartile ranges) are reported in the development study. We considered a *p* value of ≤0.05 statistically significant.

The predictive ability of the URS model was quantified by examining measures of both calibration and discrimination. Calibration was determined graphically by constructing calibration belts (package givitiR) and analytically using the Hosmer–Lemeshow test. The calibration belts reflect the agreement between predicted probabilities from the URS model with actual outcomes. With respect to other traditional approaches, they offer the possibility of detecting subgroup(s), where the disagreement between predicted probabilities and observed frequencies is significant, and the possibility of determining the direction of miscalibration [18]. Finally, calibration of the model is considered acceptable when the calibration belt encompasses the bisector in the whole 0–1 range. Discrimination was determined by calculating and plotting the AUROC curve (package pROC) and estimating the 95% confidence interval (95% CI) using stratified bootstrapping.

Furthermore, AT is one of the most important independent variables in the URS model. Due to widely reported subpopulations of PBC patients with normal or near-normal baseline AT values, we tried to separately quantify the predictive ability of the URS model in the PBC subpopulations with both normal and increased baseline AT values. Analyses were performed by a biomedical statistician in RStudio (version 1.2.1335).

## 4. Results

**Figure 1:**
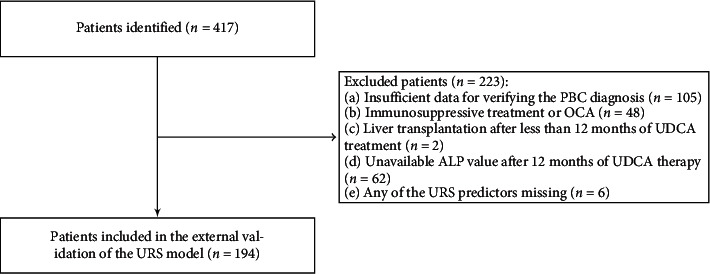
Flowchart of patient recruitment.

Four hundred seventeen patients were initially evaluated centrally by a joint committee of two study investigators, and 223 patients were excluded based on the selection criteria. We performed a complete-case analysis on 194 patients with primary biliary cholangitis (133 from Slovakia (68.56%) and 61 from Croatia (31.44%)) (Figure 1). One hundred sixty-seven patients were AMA positive (86.08%), and six patients (3.09%), both AMA and ANA negative, were diagnosed by meeting the following criteria only: (1) elevated ALP and (2) histological signs after liver biopsy.

**Figure 2:**
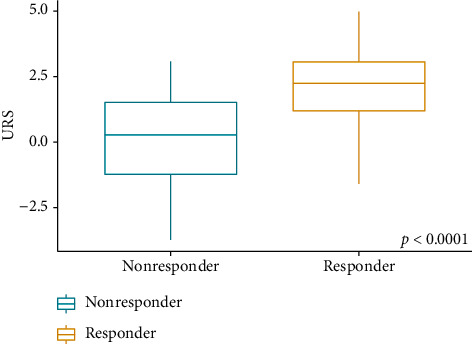
Boxplots of the ursodeoxycholic acid response score in Slovak and Croatian patients.

We report baseline clinical and demographic characteristics of both Slovak and Croatian patients together with the baseline characteristics of the derivation (UK-PBC) cohort in Table 1. Slovak and Croatian patients had lower baseline ALP and AT values than those form the UK-PBC cohort. Furthermore, 154 (79.38%) patients achieved a treatment response after 12 months of UDCA therapy (responders) compared with only 1902 (70.4%) patients in the derivation cohort (*p*=0.008). Median URS in Slovak and Croatian patients was 2.24 (IQR 1.87) in responders and 0.28 (IQR 2.74) in nonresponders (*p* < 0.0001; Figure 2). Slovak and Croatian patients were treated with a median of 1000 mg of UDCA per day (IQR 750–1250 mg per day).

**Figure 3:**
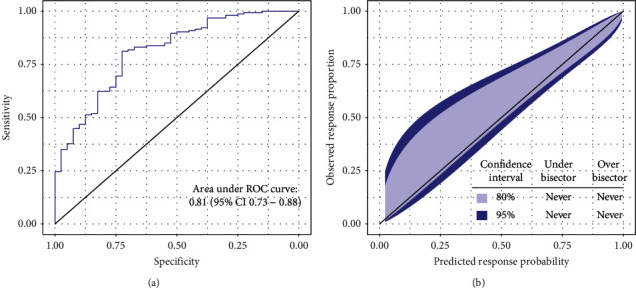
Predictive performance of the URS in Slovak and Croatian PBC patients. (a) AUROC 0.81 (95% CI 0.73−0.88) demonstrates high discrimination ability of the URS model. (b) Calibration belt confirms a well-calibrated URS model.

We confirmed a high discrimination ability of the URS model (AUROC 0.81; 95% CI 0.73−0.88) for treatment response in a combined cohort of Slovak and Croatian patients. The calibration belt revealed that the response rates were correctly estimated by the predicted probabilities. However, a slight, nonsignificant trend towards underestimating the proportion of responders was present in the lower probabilities range (Figure 3). The Hosmer–Lemeshow test showed no evidence of lack of fit to the data (*p*=0.77).

**Figure 4:**
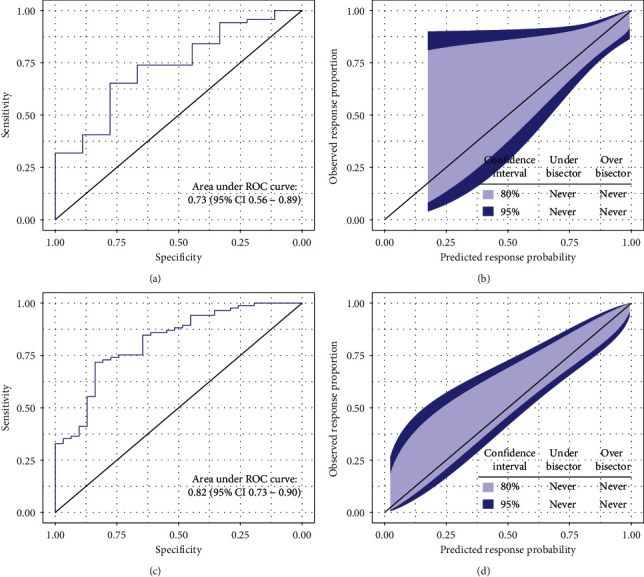
Predictive performance of the URS in patients with normal and increased baseline AT values. (a) Good discrimination ability in patients with normal baseline AT values. (b) Calibration belt in patients with normal baseline AT values characterized by wide confidence intervals in the lower predicted probabilities range. (c) Good discrimination ability in patients with increased baseline AT values. (d) Calibration belt demonstrating good calibration in patients with increased baseline AT values.

Additionally, we quantified predictive performance of the model in patients with normal (*n* = 78 (40.21%)) and increased (*n* = 116 (59.79%)) baseline AT values. Interestingly, the discrimination ability was lower in patients with normal baseline AT values (AUROC 0.73, 95% CI 0.56–0.89) compared with that in patients with increased baseline AT values (AUROC 0.82, 95% CI 0.73–0.90). Despite the presence of wide confidence intervals, the URS model was well calibrated in patients with both normal and increased AT values as the Hosmer–Lemeshow test revealed no evidence of a lack of fit to the data (*p*=0.58 and *p*=0.99, respectively) (Figure 4).

## 5. Discussion

Carbone et al. proposed the URS model to predict treatment response as defined by the Toronto criteria [13]. Although there are several distinctive definitions and continuous scoring systems of the first-line treatment response in PBC patients, the authors chose the Toronto criteria because this was how the treatment response had been defined in clinical trials of the second-line agents [12]. The URS was developed using rigorous logistic regression modelling. The authors used a cohort of PBC patients from the United Kingdom that consisted of 2703 participants and was externally validated on 984 PBC patients from Italy [13]. Further validation in other geographical regions is essential, however, to universally endorse the URS model. Our results confirm the calibration and discriminatory ability of the URS model as reported in the original study.

Yagi et al. performed the first independent external validation of the URS model on 726 Japanese patients receiving UDCA monotherapy [16]. The authors used ALT instead of the composite AT variable and applied the same treatment response definition (ALP ≤ 1.67 × ULN after 12 months of UDCA therapy). Yagi et al. evaluated the model's discrimination ability using the original and a modified URS equation. The AUROC of the original URS model was 0.77 (95% CI 0.70–0.83), and the AUROC of the modified URS model (using pretreatment data only) was 0.87 (95% CI 0.70–0.83). The authors did not report on any measures of the model's calibration.

Chen et al. proposed another model to estimate the future response to the first-line treatment in PBC patients [19]. In this case, the authors defined the treatment response based on the Barcelona criteria combined with the Paris I criteria. Although similar predictive variables were used, the reported discrimination ability was lower than these of the URS model (AUROC 0.763 (95% CI: 0.701–0.817) and 0.798 (95% CI: 0.681–0.887) in internal and external validation, respectively). The authors did not report on any measures of the model's calibration. We were not able to validate or compare the predictive performance of this model due to the inability to evaluate the treatment response as defined by Paris I criteria.

The Slovak and Croatian cohort of PBC patients has a similar prevalence of AMA negativity and concurrent AMA and ANA negativity as previously reported [20]. In our cohort, both AT and ALP values were numerically lower than in the derivation (UK-PBC) cohort. Four other studies from Western countries have reported similar baseline characteristics as those from our cohort [21–24]. The proportion of responders was also significantly different between this cohort and the UK-PBC cohort although the reasons for these differences are unclear. The delay in initiating therapy with UDCA in the UK-PBC cohort (median of 75 days) may partially explain this.

Despite the differences, our study shows that the discrimination ability and the model's calibration in the patient cohorts from Slovakia and Croatia are practically identical to these reported in the original study. However, a slight, nonsignificant trend towards underestimating the proportion of the responders is present in the lower probabilities range. This trend is not restricted to Slovakia and Croatian patients only but can be observed in the GLOBE cohort as well.

In general, we demonstrated a good predictive performance of the URS model in a population characterized by a significantly higher proportion of responders than in the UK-PBC or GLOBE cohorts. Furthermore, the evidence presented in this cohort confirms the good predictive ability of the URS model in a PBC population with numerically lower baseline values of both AT and ALP compared with those in the UK-PBC or GLOBE cohorts.

This model showed good discrimination ability, albeit lower AUROC, in the PBC subpopulation with normal baseline AT values. In these patients, the previously mentioned wide calibration belts are probably a result of a truly low proportion of nonresponders rather than poor calibration of the model.

Carbone et al. recognized that the ΔALP and treatment time lag are redundant in clinical practice, but they retained them in the model to emphasize the importance of not delaying effective treatment. In this study, we verified that omitting these variables has practically no impact on the predictive performance of the model and that individual risk profiles obtained from the URS model can be used to determine a patient's risk of no response after a 12-month course of UDCA. Treatment response evaluations should be recommended for these particular patients earlier than is currently used in clinical practice and also on a regular basis.

Our study has a few limitations. First, the study cohort was recruited retrospectively using archived data, thus creating the possibility of information bias. Second, the sample size was insufficiently large to be truly representative of the whole PBC population in these two countries.

## 6. Conclusion

We confirmed that the URS model can be used in treatment naïve PBC patients from Eastern Europe for estimating the treatment response probability after 12 months of a UDCA course.

## Figures and Tables

**Table 1:** Baseline clinical and demographic characteristics of both Slovak and Croatian and derivation cohorts.

	Validation cohort (Slovak and Croatian patients, *n* = 194)	Derivation cohort (UK-PBC)
Female patients	165/194 (85.05%)	2409/2703 (89.1)
Age at diagnosis (years)	56.00 (49.00–62.00)	56.80 (49.52–64.16)
Total bilirubin (×ULN)	0.53 (0.43–0.76)	0.53 (0.37–0.76)
Aspartate transaminase (×ULN)	1.13 (0.85–1.67)	1.40 (0.90–2.25) (AT)
Alanine transaminase (×ULN)	1.23 (0.78–1.85)	1.40 (0.90–2.25) (AT)
Alkaline phosphatase (×ULN)	1.66 (1.18–2.54)	1.85 (1.21–3.25)
Gamma-glutamyl transferase (*μ*kat/l)	4.38 (2.34-6.70)	—
Albumin (g/l)	43 (40.16–44.9)	41 (38–44)
Total cholesterol (mmol/l)	5.96 (5.24–6.80)	—
High-density lipoprotein cholesterol (mmol/l)	1.60 (1.31-1.84)	—
Low-density lipoprotein cholesterol (mmol/l)	3.63 (2.94–4.20)	—
Triglycerides (mmol/l)	1.24 (0.98–1.71)	
Ferritin (pmol/l)	66.80 (26.78–118.80)	—
C-reactive protein (mg/l)	4.16 (2.93–8.70)	—
Immunoglobulin *M* (g/l)	3.54 (2.42–5.03)	—
Glycemia (mmol/l)	5.20 (4.83–5.97)	—
Platelets (×10^9^/l)	241.00 (199.25–301.00)	—
Absolute neutrophil/lymphocyte count	1.89 (1.42–2.40)	—
Prothrombin time (INR)	0.99 (0.93–1.05)	—
Ursodeoxycholic acid dosage (mg/d)	1000 (750–1250)	—

Data are presented as median (interquartile ranges) or absolute counts (%). g/l: grams per liter, INR: international normalized ratio, mg/d: milligram per day, mg/l: milligram per liter, mmol/l: millimole per liter, *μ*kat/l: microkatal per liter, *n*: number, pmol: picomole per liter, PT: prothrombin time, and ULN: upper limit of normal.

## Data Availability

The data (in an excel file) used to support the findings of this study are available from the corresponding author upon request.

## Abbreviations

ALP: Alkaline phosphatase
ALT: Alanine aminotransferase
AMA: Antimitochondrial antibodies
AST: Aspartate aminotransferase
AT: Aminotransferase
AUROC: Area under receiver operating characteristic curve
CI: Confidence interval
CRF: Case report form
EASL: The European Association for the Study of the Liver
g/l: Grams per liter
INR: International normalized ratio
IQR: Interquartile range
mg/d: Milligram per day
mg/l: Milligram per liter
mmol/l: Millimole per liter
*n*: Number
PBC: Primary biliary cholangitis
PT: Prothrombin time
TB: Total bilirubin
UDCA: Ursodeoxycholic acid
ULN: Upper limit of normal
URS: Ursodeoxycholic acid response score
*μ*kat/l: Microkatal per liter
pmol: Picomole per liter.
